# Long loop technique with bifemoral access as salvage technique for repositioning of dislodged port catheters

**DOI:** 10.1186/s42155-022-00341-y

**Published:** 2022-12-13

**Authors:** Vincent Van den Bosch, Frédéric De Beukelaer, Peter Isfort, Sebastian Keil, Christiane K. Kuhl, Philipp Bruners, Federico Pedersoli

**Affiliations:** Department of Diagnostic and Interventional Radiology, University Hospital RWTH Aachen, Aachen, Germany

**Keywords:** Port catheter dislodgement, Port catheter repositioning, Salvage technique, Transfermoral

## Abstract

**Background:**

Repositioning of dislocated port systems’ catheters is usually performed with a pigtail catheter and/or a goose snare. In case of an inaccessible port catheter tip due to thrombosis, this classic approach may be not successful. For these cases, we describe a long loop bailout technique with bifemoral access.

**Technique:**

Via a right transfemoral access, a first attempt to reposition the dislodged port catheter using pigtail catheter and goose snare was performed. After an unsuccessful attempt and delineation of thrombosis of the catheter tip, the contralateral femoral vein was subsequently punctured and a sheath was placed. Through both vascular sheaths, pigtail catheter and goose wire were advanced distally to the catheter. The guidewire in the pigtail catheter was snared, thus creating a “Long loop” configuration. Pulling down both catheters simultaneously with improved stability allowed to detach the catheter tip from the vessel wall and replacement into the superior vena cava was possible. Refinement of catheter tip position was done using the goose snare. This technique was applied on 5 patients with dislodged port catheters in the jugular vein (2/5), the innominate vein (1/5), the subclavian vein (1/5) and the azygos vein (1/5) with a technical success of 100%. No complications were observed.

**Conclusion:**

The Long loop technique can be used as salvage approach to reposition a dislodged catheter in case of failure with pigtail catheter and goose snare.

**Supplementary Information:**

The online version contains supplementary material available at 10.1186/s42155-022-00341-y.

## Background

Central-venous port catheters are reliable devices used in patients which need long-term intravenous therapy. Mechanical complications, as for example catheter dislocation, may however occur and can lead to dysfunctionality and/or removal of the system (Kulkarni et al. 2014).

Readjusting the catheter position with a pigtail catheter with or without successive repositioning by catching the tip with a goose snare currently represent the standard approach, with a high technical success rate (Chuang et al. 2011, Bessoud et al. 2003). Nevertheless, in rare cases, in which the catheter is embedded in a (at least partially) thrombotic occluded vein branch (e.g. azygos vein), there is no free end to be caught with the goose snare and the pigtail catheter may not be stable enough to retrieve the catheter (Mori et al. 2021). Here we present a technique in which a long loop across the dislodged catheter was established with a goose snare and the guidewire after venous access via both femoral veins in order to increase the stability and reposition the port catheter.

## Material and methods

### Patient population

A retrospective analysis was conducted on all repositions of dislodged port catheters performed between 2016 and 2022 at a tertiary care center. Approval for this study was waived by the institutional review board. Written informed consent was obtained in all patients. All cases in which the long loop technique was applied after unsuccessful attempts with pigtail-catheter and/or snare were reviewed. Electronical medical records and interventional data were evaluated to obtain patient data (sex, age), signs and symptoms of dislodgment, port model, venous access, site of the dislocated catheter, material used in the repositioning, fluoroscopy time, radiation dose and intraoperative complications. Technical success was defined as the correct readjustment of the catheter position in the superior vena cava.

### Technique of treatment

Laboratory data with full blood counts, partial thromboplastin time and prothrombin time were achieved within two weeks before the intervention. All procedures were performed in an angiographic suite (Artis zeego eco or Artis zee, Siemens Healthcare). The right common femoral vein was punctured under sonographic guidance after local anesthesia (Mepivacaine 1%, PUREN Pharma GmbH & Co. KG). An 0.035-inch, hydrophilic guidewire (Terumo Corporation) was advanced in the vena cava inferior and a 5F short introducer sheath (Terumo Corporation) was inserted in Seldinger technique. Firstly, an attempt with a 5F pigtail catheter (Merit Medical Systems, Inc.) by hooking the tip of the catheter and pulling inferiorly. When unsuccessful, a second attempt was made using a 4F goose snare catheter (Andramed GmbH) to grab the tip of the dislodged catheter. Because of attachment of the catheter tip due to thrombotic material, the tip could not be mobilized with the pigtail catheter and was not accessible by the goose snare.

**Fig. 1:**
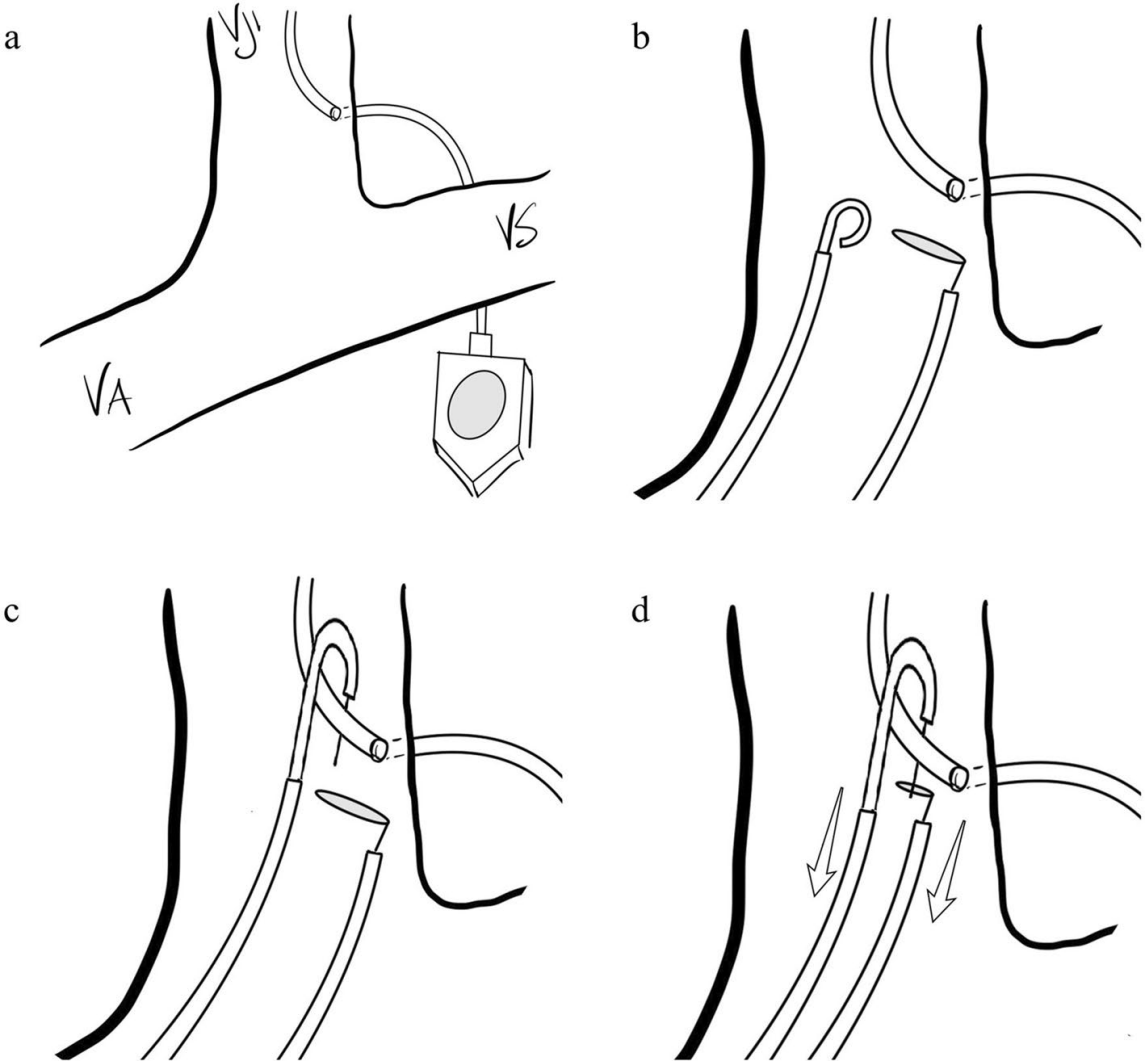
Schematic drawing of the “Long loop technique”. **a** A port-a-cath inserted via the left jugular vein, with cranially dislodgement of the catheter in the same vessel. **b** Advancement of both a pigtail catheter and a goose snare. **c** Hooking of the dislodged catheter with the pigtail catheter, also advancement of a guidewire in the pigtail catheter. **d** Catching of the guidewire with the goose snare, creating a “long loop” configuration. Repositioning by pulling the catheters caudally

**Fig. 2:**
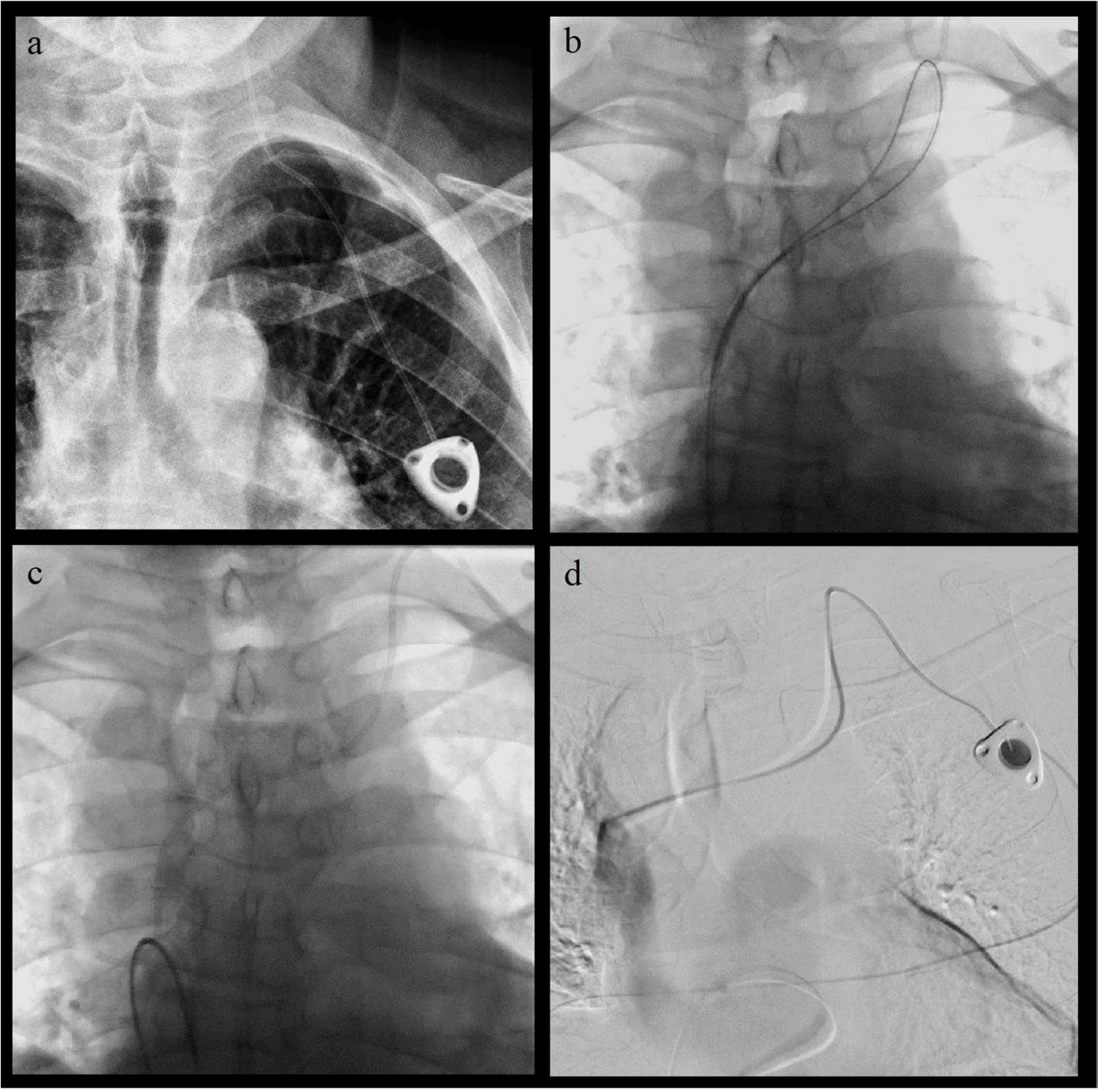
Clinical sample case. **a** X-ray showing the port systems inserted in the left jugular vein with catheter dislodged high in the ipsilateral jugular vein. **b** Fluoroscopic image of the long loop with partial reposition of the port catheter in the innominate vein. **c** Fluoroscopic image with almost complete reposition of the port catheter. **d** Digital subtraction angiography proving the functionality of the port system

Subsequently, a contralateral venous access was established in the above-mentioned technique. A “long loop” configuration was created by advancing both the used pigtail and the goose snare catheter distally to the neck of the dislodged port catheter and snaring the tips of the hydrophilic guidewire introduced through the pigtail catheter with the goose snare (Figs. 1 and 2). Both catheters were then pulled down simultaneously to reposition the dislodged port catheter in the vena cava superior with increased stability (see video in the supplementary material). The goose snare was then used to catch and adjust the position of the port catheter position. Next, the port system was punctured with a non-coring needle and 10 ml of diluted contrast agent (Ultravist 300, Bayer AG) were injected to test its functionality. In case of thrombosis, 2 mg Alteplase (Actilyse, Boehringer Ingelheim) were applied and the catheter was controlled the day after. All materials were removed and both inguinal puncture sites were manually compressed until hemostasis.

## Results

Between 2016 and 2022, a total of 1883 patients received a port-a-cath in our department. In the same period of time. Ninety-four catheter corrections were performed. Of these group of patients, a total of 5 patients (m/f: 4/1, mean age 58.6 ± 6.9 y.o.) were treated by means of the technique described above between 2016 and 2022. No direct causes for the dislodgments could be identified: the position and length of the catheter and puncture site were considered correct in every of these patients. Observation of migration of the catheter-tip was after a mean of 498 days (range 61 – 1822 days) after implantation. Symptoms of presentation were irrigation resistance in all patients. One single patient, with a dislodged catheter into the left internal jugular vein, reported a feeling of pressure in the left ear. Technical success was obtained in all 5 patients (100%), with need for transcatheter thrombolysis in one single patient (20%). No complications were observed. Mean fluoroscopy time was 36:49 ± 11:05 min with a mean dose area product (DAP) of 3088 ± 1589.5 cGycm^2^.

## Discussion

This technique showed to be effective and safe in repositioning dislodged catheters, so avoiding removal and potential reimplantation of the port system.

A recent study used a similar maneuver to remove a fragment of a central venous catheter which migrated in the right atrium, with both ends in the atrial appendage and thus not easily accessible for the goose snare (Mori et al. 2021). Similarly, other recent studies created a long loop to mobilize a free end of a fractured port catheter into the inferior cava vein, which was located across the right atrium and ventricle and consequently difficult to be caught with a snare (Haga et al. 2020, Shah et al. 2022). In our cohort, we used the long loop to reposition, not to retrieve, a port catheter which was dislodged and embedded in thrombotic material and consequently not accessible for the snare. The usage of pigtail catheter could not provide enough force to mobilize the port catheter, which remained blocked in the side vein. For the described approach, we decided to use two short 5F introducer sheath instead of a long 10F introducer sheath as described by all these authors to be able to move the two catheters independently and thus to increase the freedom of movement of the long loop.

Possible limitations of this technique are represented by the quite long fluoroscopy time and the consequently high required amount of radiation dose. Also, a second operator or assistant to pull the contralateral catheter is needed. Moreover, the costs of this technique can be comparable or higher compared to the removal and the placement of a new system. Nevertheless, the decision to proceed with this maneuver was taken during the intervention, in which already multiple materials were already used. At this time of the intervention, deciding to remove the port and place a new catheter could be even less cost effective. In general, the alternative solution, removal of the port-a-cath and implantation of a new one, may have caused an increased discomfort for the patient and with the risks inherent to a repeated procedure. The decision should be taken after talking with the patient and discussing and considering both treatment options.

A similar technique has also been described to retrieve other materials such as inferior vena cava filters (Najafi et al. 2018, Al-Hakim et al. 2015).

## Conclusions

This “long loop technique” is a valid alternative strategy in case of failure of port catheter repositioning retrieval with pigtail catheter or goose snare.

## Supplementary Information

**Additional file 1**.

## Data Availability

The datasets analysed during the current study are available from the corresponding author on reasonable request.

## Abbreviation

DAP: Dose area product
